# Genomic Variants, Transcriptomic Profile, Ultrasonographic Findings, and Antioxidant and Immunological Biomarkers Linked to Pregnancy Toxemia Susceptibility in Goats

**DOI:** 10.3390/vetsci12090891

**Published:** 2025-09-15

**Authors:** Ahmed El-Sayed, Mohamed Marzok, Huda A. Alqahtani, Amin Tahoun, Adel I. Almubarak, Rasha Yassin Elkhidr, Zakriya Al Mohamed, Elshymaa A. Abdelnaby, Hussein Babiker, Hanan M. Alharbi, Khairiah M. Alwutayd, Ahmed Ateya

**Affiliations:** 1 Department of Animal Health and Poultry, Animal and Poultry Production Division, Desert Research Center (DRC), Cairo 11753, Egypt; 2 Department of Clinical Studies, College of Veterinary Medicine, King Faisal University, Al Ahssaa 31982, Saudi Arabia; 3 Department of Zoology, College of Science, King Saud University, P.O. Box 2455, Riyadh 11451, Saudi Arabia; hudalqahtani@ksu.edu.sa; 4 Department of Animal Medicine, Faculty of Veterinary Medicine, Kafrelshkh University, Kafrelsheikh 33516, Egypt; 5 Department of Clinical Veterinary Medical Sciences, Jordan University of Science and Technology, Irbid 22110, Jordan; 6 Department of Biology, College of Science, Princess Nourah bint Abdulrahman University, P.O. Box 84428, Riyadh 11671, Saudi Arabia); 7 Department of Development of Animal Wealth, Faculty of Veterinary Medicine, Mansoura University, Mansoura 35516, Egypt

**Keywords:** goats, pregnancy toxemia, gene expression, immunity, antioxidant, nucleotide sequence variants, ultrasound

## Abstract

Pregnancy toxemia (PT) represents a significant metabolic disorder affecting small ruminants that causes substantial economic losses due to reduced productivity, reproductive failure, and high mortality. This study investigated the clinical, ultrasonographic, hematobiochemical, oxidative stress, and immunological profiles, as well as the gene expression and nucleotide sequence variations, associated with PT susceptibility in Shami goats. Fifty late-pregnant does (33 healthy and 17 PT-affected) were examined. Clinical evaluation, complete blood count, biochemical analysis, cytokine profiling, antioxidant assays, hepatic ultrasonography, quantitative real-time PCR of immune (IL6, IL8), antioxidant (SOD3, HMOX1), and lipogenic (ACACA, FASN) genes, and PCR-DNA sequencing were performed. PT does exhibit significant hypoglycemia, hyperketonemia, elevated liver and kidney function biomarkers, dyslipidemia, oxidative stress (↑ MDA, ↓ GSH, GPx, SOD, CAT), increased pro-inflammatory cytokines (IL1α, IL1β, IL6, TNFα), and reduced IL10. Gene expression analysis revealed upregulation of IL6 and IL8 and downregulation of SOD3, HMOX1, ACACA, and FASN in PT does. Sequencing identified multiple synonymous and non-synonymous SNPs significantly associated with PT. Ultrasonography indicated hepatic fatty infiltration. Discriminant analysis using SNPs achieved 100% classification accuracy between healthy and PT does. These findings suggested that combined clinical, biochemical, oxidative, immunological, and genetic markers could enhance early PT diagnosis and may provide a basis for future studies aimed at selective breeding for improved resistance.

## Simple Summary

Pregnancy toxemia is a life-threatening metabolic disease in late-pregnant goats, leading to severe health problems, reproductive losses, and high mortality. This study examined Shami goats to identify clinical signs, blood changes, liver ultrasound patterns, and genetic markers linked to the disease. Affected goats showed anemia, liver and kidney dysfunction, oxidative stress, and increased inflammation. Specific gene expression patterns and DNA variations were strongly associated with susceptibility. These findings support using combined clinical, biochemical, and genetic markers for early diagnosis and selective breeding to reduce disease impact and improve goat herd health.

## 1. Introduction

Goats are a vital resource for both the economy and culture, particularly in developing regions where they provide meat, milk, fiber, and hides [1]. Their resilience to challenging environments makes them especially valuable in semi-arid and desert areas, where they contribute substantially to livestock-dependent livelihoods. Egypt alone maintains around 4.2 million heads [2]. Among these, the Damascus (Shami) goat is highly prized across the eastern Mediterranean because of its outstanding reproductive efficiency and production capacity [3]. Its adaptability to hot climates and lowland pastures gives it a reproductive advantage over sheep in arid settings [4]. Owing to its superior genetics and dual-purpose production, this breed is often employed in crossbreeding programs to improve the milk and meat output of local goat populations [5].

The periparturient phase, covering roughly three weeks before until three weeks after kidding, is a decisive period that affects both present and future productivity in farm animals [6]. Inadequate physiological adjustment during this window predisposes animals to metabolic disorders [7]. Pregnancy toxemia (PT) is the most frequent metabolic disturbance in small ruminants during late gestation. It is primarily driven by negative energy balance (NEB), leading to hypoglycemia and elevated ketone bodies [8]. The condition generally arises in the last weeks or days of pregnancy, with a reported prevalence of 5–20% and mortality rates as high as 80% if untreated [9,10,11]. Even when therapeutic measures such as fluid replacement, glucose supplementation, electrolyte correction, or propylene glycol are used, losses can remain high, with mortality still reaching 40%. Survivors are frequently left with adverse outcomes, including premature parturition, abortion, or stillbirth [12]. PT results from inadequate glucose availability to meet the demands of rapidly developing fetuses. A wide range of risk factors including nutritional status, metabolic and genetic predisposition, management practices, and parity play roles in disease development [13,14]. Animals reared under intensive systems, carrying multiple kids, or of older age are particularly vulnerable. Clinical signs often include reduced feed intake, rapid breathing, ruminal stasis, vision disturbances, lethargy, drooping ears, abnormal posture of the head and neck, metabolic acidosis, fatty liver infiltration, hypocalcemia, and hyperketonemia [15]. Because of its high mortality, timely detection is essential, although early manifestations can be vague and difficult to recognize [8]. A combination of routine biochemical profiling and clinical assessment is therefore regarded as central to early diagnosis [16].

Biochemical blood analysis provides insight into nutritional balance, health status, and physiological function and thus plays an important role in herd-level monitoring [17,18]. In particular, blood β-hydroxybutyric acid (BHBA) is considered the reference marker for detecting both clinical and subclinical PT [19]. Variations in biochemical indicators beyond normal ranges also signal tissue damage [20]. Furthermore, oxidative stress is a frequent consequence of imbalance between free radical generation and antioxidant defenses [21]. Animals rely on protective mechanisms such as enzymatic antioxidants (e.g., catalase) to neutralize reactive species [22,23]. Cytokines also play a central role in regulating immune and metabolic responses. Produced locally in response to stimuli, these signaling molecules act in paracrine, autocrine, or endocrine fashions but typically have short half-lives [24].

With the expansion of molecular genetics, new avenues have opened to strengthen disease prevention and improve livestock health [25]. Genetic markers associated with resistance or vulnerability to disease have been identified in cattle [26], demonstrating that susceptibility can differ among host genomes [27]. Yet, for goats and specifically the Damascus breed, information remains scarce regarding the biochemical, antioxidant, and immune alterations involved in PT, as well as the gene expression changes and single-nucleotide polymorphisms (SNPs) linked to disease risk. Moreover, no previous investigations have simultaneously explored transcriptomic patterns alongside SNP markers to clarify the genetic basis of PT in Shami goats. This study was therefore designed to integrate gene expression profiling with SNP detection, combined with immunological, antioxidant, and biochemical evaluation, in order to provide a more complete understanding of PT susceptibility in this breed.

## 2. Materials and Methods

### 2.1. Animals and Study Design

**Figure 1:**
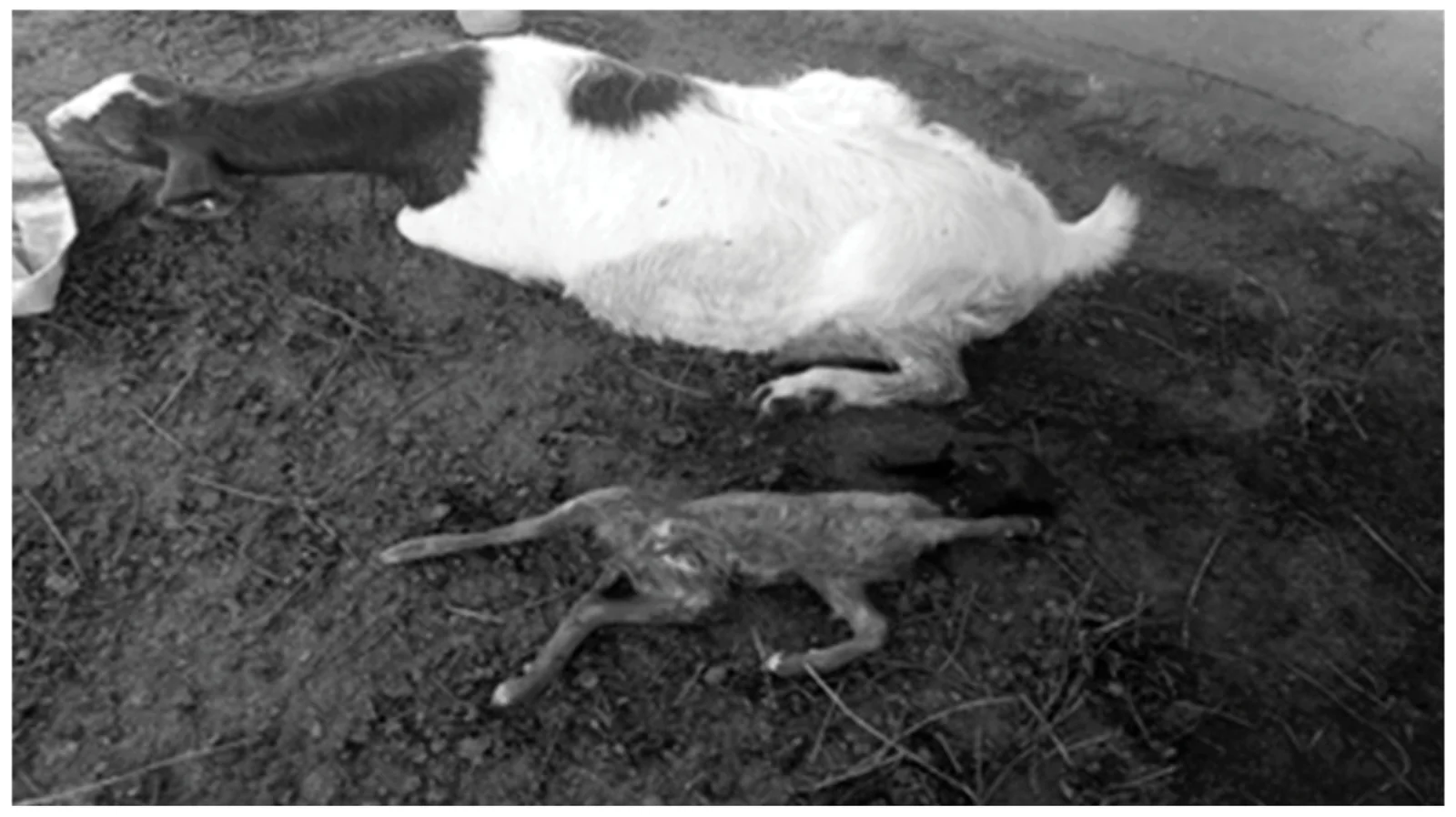
A doe affected by pregnancy toxemia, positioned in sternal recumbency. The animal displayed characteristic signs of late-stage pregnancy toxemia, including marked weakness and lethargy. The pregnancy resulted in a single non-viable fetus, illustrating the severe effects of the disorder on both maternal health and fetal viability.

This study was conducted on 50 late-pregnant Shami goats (gestational age 120–135 days; full-term ~150 days) with a mean age of 4.9 ± 0.7 years and an average body weight of 49.16 ± 6.5 kg. The does were in their 4th to 5th parity, and body condition scores (BCSs) ranged between 2.5 and 3.5 (on a 5-point scale). Pregnancy was confirmed using a Samsung Medison SONOACE R3 ultrasound machine (Samsung Medison Co., Ltd., Cheonho-daero, Gangdong-gu, Seoul, Republic of Korea) after natural mating. The experiment took place at the Mariut Research Station, Desert Research Center, El-Amria, Alexandria, Egypt, during the late gestational season (March–April). At this time of year, the average ambient temperature ranged between 22 and 28 °C, with relative humidity levels of 50–60%. The goats were housed in semi-open shaded pens that allowed natural ventilation, minimizing heat stress and ensuring welfare during the study period. Each doe received a daily ration consisting of 750 g of concentrate feed mixture (CFM) and 750 g of alfalfa hay, with the formulation of the basal diet presented in Table 1, as well as 200 g of natural pasture consisting of grass, berseem, darawa, and other green herbs. This ration was designed to meet both maintenance and production requirements of late-gestation goats in accordance with [28] nutrient recommendations. Of the animals examined, 33 were clinically healthy does, and 17 were diagnosed with pregnancy toxemia (PT). Goats were classified into pregnancy toxemia (PT) and healthy groups based on clinical examination and biochemical results. Control group inclusion criteria were as follows: β-hydroxybutyric acid (β-HBA) ≤ 0.8 mmol/L [14], normal vital parameters (temperature, pulse, respiratory rate), absence of ocular or nasal discharge, bright eyes, normal appetite and mobility, and unremarkable parturition history. PT group criteria included β-HBA > 2.5 mmol/L and the presence of confirmed clinical signs such as anorexia, hypothermia, polypnea, ruminal atony, blindness, depression, stiffness, incoordination, drooping ears and neck, bruxism, occasional constipation, poor body condition, acetone odor on the breath, and dystocia. Among the PT does, four aborted twins and six aborted a single fetus (Figure 1).

A comprehensive clinical assessment was performed on the does, including the measurement of the vital parameters heart rate, body temperature, and respiratory rate according to the method described by [29]. These data were recorded simultaneously. The animals had ad libitum access to clean drinking water and were housed in semi-open, shaded pens. Each doe received a daily ration consisting of 750 g of concentrate feed mixture (CFM) and 750 g of alfalfa hay, with the formulation of the basal diet presented in Table 1. When available, natural pasture comprising grass, berseem, darawa, and other green herbage was also provided. In compliance with the Egyptian Authority Program, all animals were regularly vaccinated and dewormed.

#### Blood Sampling

Approximately 10 mL of blood was collected from each doe via jugular venipuncture at ~8:00 AM, 10–15 days before expected parturition. Samples were divided into plain tubes (for serum) and EDTA tubes (for whole blood). Tubes were immediately chilled on ice and transported to the laboratory. Serum was obtained by centrifugation (3000 rpm, 15 min), aliquoted, and stored at −20 °C. Whole blood was used for complete blood count (CBC) and RNA extraction.

### 2.2. Ultrasonographic Examination

All 50 does underwent transcutaneous ultrasonography (Samsung Medison SONOACE R3 with 2–8 MHz convex probe, Cheonho-daero, Gangdong-gu, Seoul) around 10–15 days prepartum, in parallel with blood collection. The examination was carried out during late gestation, approximately 10–15 days before expected parturition, in parallel with blood sampling. The animals were allowed to stand during imaging, and the captured images were stored for later analysis. The right side of each doe was shaved from the ventral abdomen to the transverse processes of the vertebrae, extending to a handbreadth posterior to the last rib. Each intercostal space (ICS) was scanned with the transducer positioned parallel to the ribs, moving from dorsal to ventral after the application of ultrasound transmission gel (Ultra-Gel, Medi Lab Industry, Cairo, Egypt). The echotexture of the liver, caudal vena cava, and portal veins was initially evaluated. Interpretation of fatty liver infiltration was based on ultrasonographic appearance; histopathological confirmation was not performed due to the non-invasive design of the study.

### 2.3. Total RNA Extraction, Reverse Transcription, and Quantitative Real-Time PCR

Total RNA was extracted from blood using Trizol combined with the RNeasy Mini Kit (Qiagen, Cat. No. 74104, Waltham, MA, USA, USA), following the supplier’s guidelines. RNA yield and purity were verified with a NanoDrop ND-1000 spectrophotometer (Quawell, USA). One microgram of RNA was reverse transcribed into cDNA with the RevertAid First Strand Kit (Thermo Fisher, Cat. No. EP0441, London, UK). Gene expression was quantified via SYBR Green qRT-PCR (SensiFast™ SYBR, Bioline, Cat. No. Bio-98002, London, UK) targeting immune markers (IL-6, IL-8), antioxidant genes (SOD3, HMOX1), and lipogenic genes (ACACA, FASN). Primer sequences were designed from Capra hircus references in GenBank (Table 2). β-actin served as the internal control due to its consistent expression in goat blood across physiological conditions [30,31].

Each 25 µL PCR reaction included 8.25 µL of RNase-free water, 0.5 µL of each primer, 0.25 µL of reverse transcriptase, 3–4 µL of 5 × buffer, and 12.5 µL of master mix. The cycling profile included 30 min at 50 °C (reverse transcription), 10 min at 94 °C (initial denaturation), and 40 cycles of 94 °C (15 s), gene-specific annealing (1 min), and 72 °C (30 s). A melting curve was run to confirm specificity. Relative expression was calculated by the 2^−ΔΔCt^ method [32] using the healthy group as calibrator.

### 2.4. DNA Sequencing and SNP Analysis

PCR products of expected sizes were purified (Jena Bioscience Kit, Cat. No. PP-201×S, Germany) [33], Concentration and quality were checked with a NanoDrop spectrophotometer (Q5000, Waltham, MA, USA) [34]. Sequencing was carried out bidirectionally using the enzymatic chain termination method on an ABI 3730XL sequencer (Applied Biosystems, USA) [35].

Sequences were processed with Chromas 1.45 and BLAST 2.0 [35]. Single-nucleotide polymorphisms (SNPs) were identified by aligning samples with the Capra hircus ARS1 reference genome (NCBI GCF_001704415.1) using MEGA6 software version 6 [36]. SNPs located within coding regions were examined for amino acid substitutions. Associations between variants and PT status were determined by comparing allele/genotype frequencies between healthy (*n* = 33) and affected (*n* = 17) groups

### 2.5. Biochemical, Immunological, and Antioxidant Parameters

Serum samples (collected as described in the Blood Sampling section) were analyzed for biochemical, immunological, and antioxidant parameters. All biochemical, immunological, and antioxidant parameters were measured from serum samples obtained during late gestation, approximately 10–15 days before expected parturition. These analyses were performed on all 50 does included in the study (33 healthy and 17 PT-affected). Commercial diagnostic kits were used according to the manufacturers’ instructions. Serum analyses were performed using commercial kits from Spectrum Company Cairo, Egypt) on a selective chemistry analyzer (Apple 302, Cupertino, CA, USA). Globulin levels were calculated by subtracting albumin from total protein. Beta-hydroxybutyrate (BHB) was measured with a kit from Cayman Chemical (USA; Item No. 700190), and non-esterified fatty acids (NEFA) were determined using a kit from Randox Laboratories Ltd. (Crumlin, Co. Antrim, London, UK). Serum malondialdehyde (MDA) and antioxidant enzymes—including catalase (CAT), glutathione peroxidase (GPx), and glutathione reductase (GSH)—were quantified spectrophotometrically using kits from Biodiagnostic Company, Cairo, Egypt^®^. Levels of pro-inflammatory cytokines (IL-1α, IL-1β, IL-6, TNF-α) and the anti-inflammatory cytokine IL-10 were measured using ELISA kits from MyBiosecure Company, Giza, Egypt^®^.

### 2.6. Statistical Analysis

Data were processed with SPSS (version 20 and 23, Armonk, NY, USA). Independent-sample *t*-tests compared hematological, biochemical, immunological, antioxidant, and gene expression results between groups. SNP associations with PT status were assessed by chi-square (χ^2^) or Fisher’s exact test, with odds ratios (ORs) and 95% confidence intervals (CIs) calculated. Results are expressed as mean ± SD. Linear Discriminant Analysis (LDA) was applied to evaluate whether SNP profiles of the six investigated genes could classify animals into PT or healthy groups. Gene scores were used as predictors, and health status was used as the grouping variable. Significance was set at *p* < 0.05.

## 3. Results

### 3.1. Clinical Findings

Pregnancy toxemia (PT) does exhibited marked alterations in vital signs compared with healthy controls. Body temperature and pulse rate were significantly reduced (*p* < 0.05), whereas respiratory rate was elevated. Mean values were 37.2 ± 0.1 °C, 52.3 ± 1.4 beats/min, and 37 ± 0.5 breaths/min, respectively, in the PT group, compared with 39.1 ± 0.05 °C, 84.3 ± 2.3 beats/min, and 27 ± 0.5 breaths/min in healthy does (Table 3).

**Figure 2:**
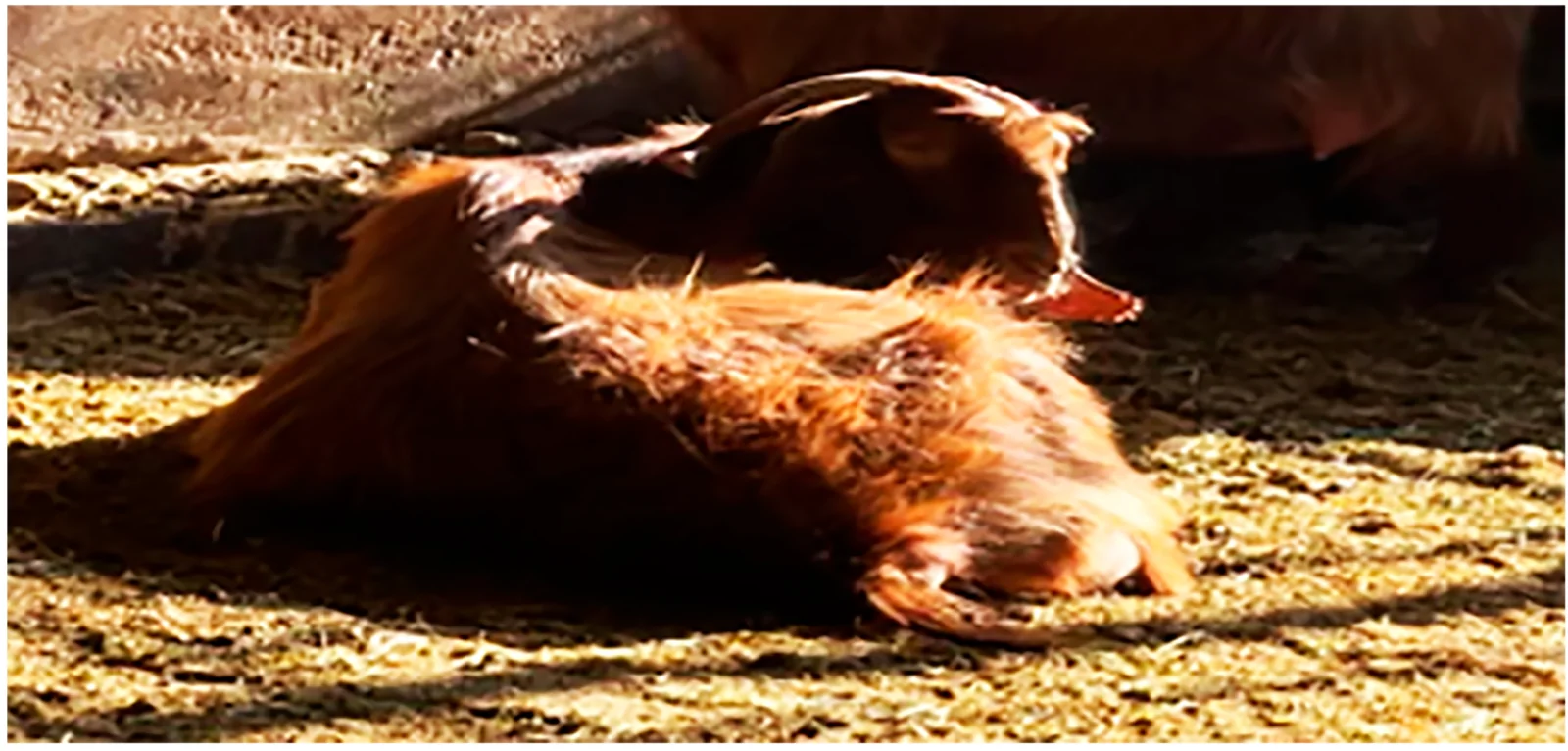
A doe with pregnancy toxemia showing neurological abnormalities. The animal exhibited convulsions, tremors in the neck muscles, and loss of vision, illustrating the effects of severe metabolic imbalance on the nervous system during late pregnancy.

The most frequent clinical signs among PT animals included depression, restlessness, constipation, wasting, acetone odor on the breath, dystocia, lateral recumbency, convulsions, muscular tremors of the neck, vision loss, stiffness, and incoordination (Figure 2).

### 3.2. Ultrasonographic Findings

**Figure 3:**
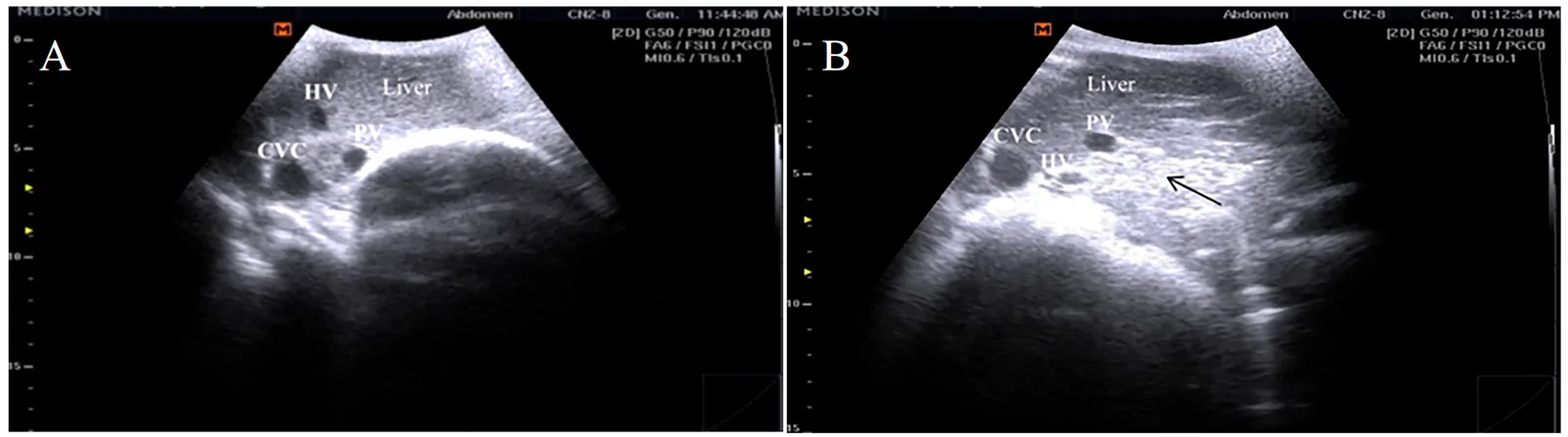
Ultrasound examination of the liver in does. (**A**) Healthy doe: scan from the 10th intercostal space (ICS) on the right side showing normal liver echogenicity and a homogeneous hepatic structure with numerous weak echoes evenly distributed. PV: portal vein; HV: hepatic vein; CVC: caudal vena cava. (**B**) Pregnancy toxemic (PT) doe: scan from the 10th ICS on the right side showing fatty liver infiltration, indicated by hyperechoic areas (black arrow) with increased parenchymal brightness and echogenicity, while hepatic vessels remain visible. PV: portal vein; HV: hepatic vein; CVC: caudal vena cava.

In healthy does, hepatic scans displayed uniform echogenicity with evenly distributed echoes across the parenchyma (Figure 3A). In contrast, PT animals showed hepatic steatosis characterized by increased brightness and echo density of the liver parenchyma while hepatic vessels remained visible (Figure 3B).

### 3.3. Patterns for Transcript Levels of Immune, Antioxidant, and Lipogenic Indicators

**Figure 4:**
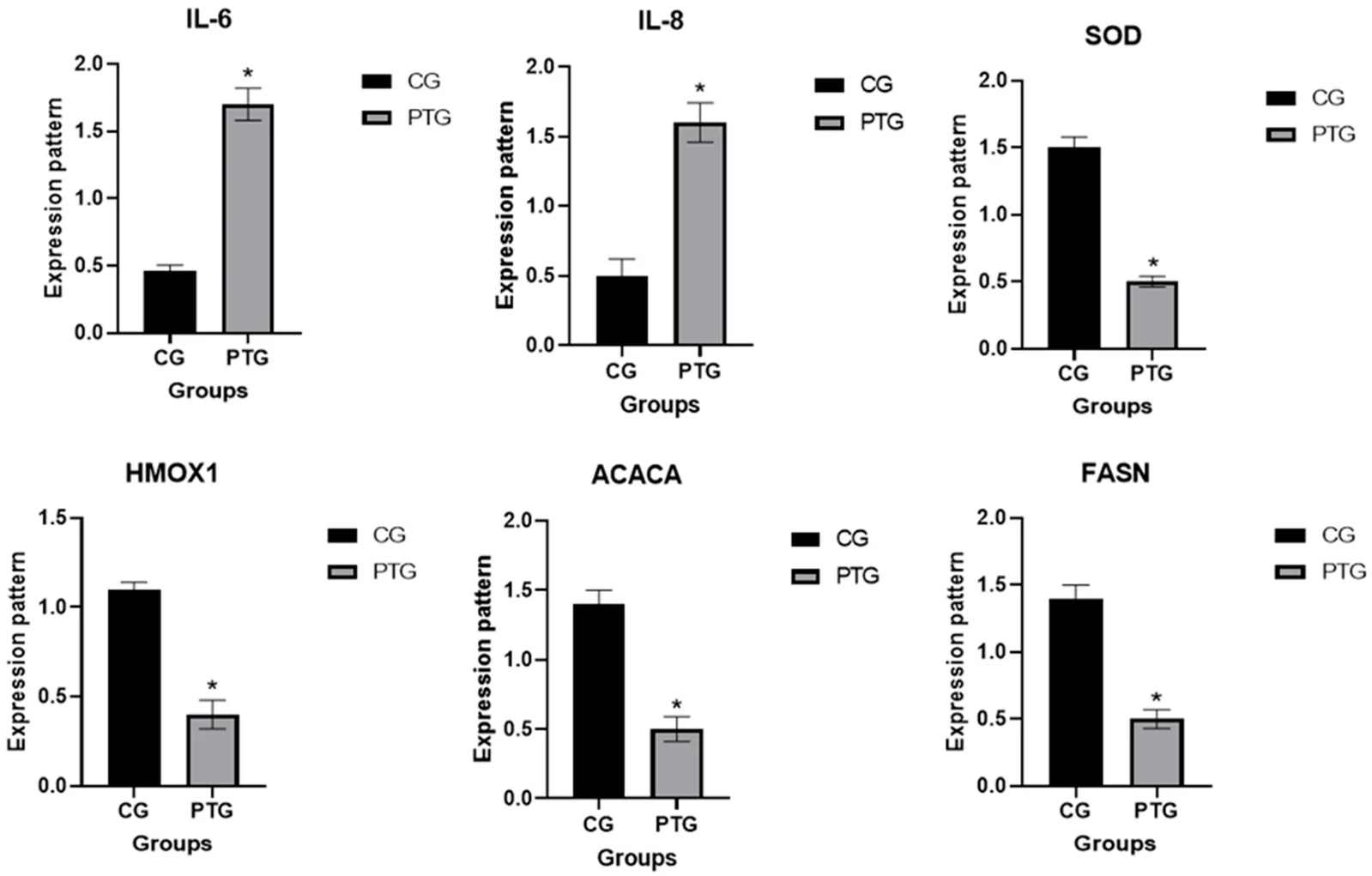
Relative mRNA expression (fold change ± 95% CI) of immune (IL-6, IL-8), antioxidant (SOD3, HMOX1), and lipogenic (ACACA, FASN) genes in healthy and pregnancy toxemic (PT) does. Expression levels were normalized to β-actin and calculated using the 2^−ΔΔCt^ method. * *p* < 0.05.

As shown in Figure 4, pregnancy toxemia (PT) does exhibit significantly elevated expression of IL-6 and IL-8 compared with healthy controls, with fold changes of 3.8 (95% CI: 2.9–4.7) and 3.1 (95% CI: 2.4–3.9), respectively. In contrast, SOD3, HMOX1, ACACA, and FASN expression was downregulated, with fold changes of 0.42 (95% CI: 0.31–0.54), 0.37 (95% CI: 0.28–0.49), 0.46 (95% CI: 0.34–0.59), and 0.51 (95% CI: 0.39–0.64), respectively, relative to healthy does. Expression values are presented as relative fold changes (2^^−ΔΔCt^) compared with healthy controls.

### 3.4. Genetic Polymorphisms of Immune, Antioxidant, and Lipogenic Genes

PCR-DNA sequence analysis revealed that the amplified fragments of IL-6 (627 bp), IL-8 (264 bp), FASN (381 bp), SOD3 (393 bp), HMOX1 (460 bp), and ACACA (477 bp) differed between healthy goats and those affected by pregnancy toxemia (PT). Comparison of the coding sequences between PT and healthy does (Table 4) indicated that all assessed immunological, antioxidant, and lipogenic genes exhibited alterations within their exonic regions. A total of seven synonymous and four non-synonymous single-nucleotide polymorphisms (SNPs) were identified. The frequencies of all detected SNPs differ significantly between PT and healthy does (*p* < 0.005). Chi-square analysis confirmed significant differences in the distribution of these SNPs across all genes between affected and resistant animals (*p* < 0.05) (Table 4).

Linear Discriminant Analysis (LDA) correctly classified healthy and PT does with 100% accuracy within this dataset (Table 5). However, this result should be interpreted cautiously given the limited sample size, and further validation in larger and independent goat populations is warranted.

### 3.5. Hematological, Biochemical, Immunological, and Antioxidant Profile

Compared with healthy does, PT animals exhibited significant reductions in erythrocytic indices (RBC, Hb, HCT, MCV, MCH, and MCHC), while total leukocytes, neutrophils, and lymphocytes were elevated (*p* < 0.05; Table 6).

Biochemical assays showed increases in serum AST, ALT, triglycerides, total protein, albumin, globulin, urea, creatinine, NEFA, and BHBA in the PT group, whereas glucose, cholesterol, HDL-C, and LDL-C concentrations were significantly lower (*p* < 0.05; Table 7).

Analysis of oxidative stress markers revealed that malondialdehyde (MDA) levels were significantly increased, whereas glutathione (GSH), glutathione peroxidase (GPx), catalase (CAT), and superoxide dismutase (SOD) levels were significantly decreased in pregnancy toxemia (PT) does compared with healthy controls (Table 8). Additionally, serum concentrations of pro-inflammatory cytokines IL-1α, IL-1β, IL-6, and TNF-α were significantly elevated (*p* < 0.05), while the anti-inflammatory cytokine IL-10 was significantly reduced (*p* < 0.05) in PT does relative to controls (Table 8).

## 4. Discussion

This study represents the first comprehensive attempt to integrate genomic, transcriptomic, biochemical, and ultrasonographic markers to explore pregnancy toxemia (PT) in Shami goats. Our findings identified SNPs significantly associated with disease risk, demonstrated altered expression of immune (IL-6, IL-8), antioxidant (SOD3, HMOX1), and lipogenic (ACACA, FASN) genes, and confirmed hepatic steatosis via ultrasonography. Together, these results highlight a set of candidate biomarkers that may aid in early diagnosis and support selective breeding strategies aimed at PT resistance.

Sequencing revealed eleven SNPs across the studied genes, four of which were directly associated with PT. Among these, two were non-synonymous substitutions predicted to modify protein structure and function. Non-synonymous mutations often alter protein conformation or activity, potentially impairing metabolic and immunological pathways [38,39]. In contrast, synonymous substitutions are traditionally considered less impactful, although some can influence gene regulation [38]. In the present study, substitutions such as SOD3 (L82P) and IL-8 (D66N) were predicted to reduce antioxidant and immune functions, while FASN (A72T) and ACACA (G56R) may hinder lipid synthesis, thereby exacerbating the negative energy balance central to PT. These loci warrant further functional validation [40].

Clinically, PT animals displayed anorexia, depression, weakness, acetone odor, bruxism, neuromuscular spasms, and lateral recumbency. Similar manifestations have been documented by other studies [41,42,43,44,45,46], reflecting the severe metabolic imbalance, ischemia, and hypoglycemia underlying the syndrome [47].

In sheep and goats, ultrasound has been shown to be useful for the early detection and diagnosis of a number of thoracic and abdominal disorders [7,48,49]. Ultrasonography confirmed fatty infiltration of the liver in affected goats, consistent with previous reports in goats [44,50,51], sheep [46,52], and cattle [53,54].

At the transcriptomic level, PT does exhibited upregulation of IL-6 and IL-8 alongside suppression of antioxidant and lipogenic genes. To our knowledge, this is the first report linking these markers to PT in goats, suggesting that altered gene expression may precede clinical signs. Similar trends have been noted in other ruminant disorders involving hypoxia and impaired placental function, where inflammatory cytokines are elevated while angiogenic and metabolic genes are suppressed [55].

Cytokines, including IL-6 and IL-8, are commonly recognized as indirect indicators of inflammatory responses [56]. Endogenous antioxidant status can be assessed through enzymatic and non-enzymatic defense systems of the body, such as the activity of superoxide dismutase (SOD) [57]. Additionally, heme oxygenase (HMOX) serves as a key regulatory enzyme in the heme degradation pathway, catalyzing the conversion of heme into equimolar amounts of biliverdin, carbon monoxide (CO), and free iron [58].

Although discriminant analysis achieved 100% classification accuracy in distinguishing PT from healthy does, this result should be interpreted with caution due to the relatively small dataset. While leave-one-out cross-validation supported the robustness of the result, external validation in larger and independent cohorts is required before firm conclusions can be drawn regarding the predictive utility of these SNP markers.

A sound understanding is required to identify the causes of variations in the expression of genes correlated to lipogenesis and adipogenesis processes in diverse livestock breeds [59]. Genetic selection for livestock adaptation to harsh conditions can be enhanced by the variation in the gene expression of numerous regulatory enzymes of the intermediate metabolism [60]. The lipid metabolism enzymes ACACA, FASN, and SCD have been investigated in rams [60], lactating sheep [61], and sheep of various breeds [62] under varying degrees of diet limitation. They have also been investigated in sheep under various stressful situations, such as tail docking [15,63]. Under total feed deprivation, ref. [64] identified the gene expression patterns of FASN and ACACA in pregnant Barki sheep. When the gene expression pattern was compared to that before feed deprivation, there was a notable downregulation.

Pregnancy toxemia is associated with a persistent, low-grade inflammatory condition, characterized by elevated concentrations of circulating free fatty acids and the recruitment of macrophages. These macrophages, once activated, release inflammatory mediators into the local tissue environment, thereby amplifying the inflammatory response [65,66,67]. These impacts are further intensified when inflammatory cytokines like TNFα, IL1β, and IL6 are released [55]. A state of negative energy balance is frequently followed by pregnancy toxemia, which can lead to poor gluconeogenesis, hypoglycemia, fat mobilization, ketonemia, and ultimately ketonuria [13]. It is noteworthy that the genes examined in prenatal toxicity were linked to multiple biological processes [55]. Placental vascularization (regulation of angiogenesis: blood vessel development, exocytosis and apoptosis, and involvement of interleukins, endothelial growth factors, insulin-like growth factors, and adipokines), pregnancy toxicity (regulation of metabolic process: carbohydrate and glucose metabolism and catabolic process), and hypoxic condition (regulation of nitric oxide synthase and hypoxia) were all explained by biological functions [55]. The previously outlined findings may account for the observed alterations in the expression profiles of immune-related, antioxidant, and lipogenic genes. Concerning the hematological alterations, the marked reduction in the levels of RBCs, Hb, PCV, MCV, MCH, and MCHC in PT does was consistent with findings of other studies [27,51], but differed from the findings reported by the authors of [42,46,68], who demonstrated that Hb level did not significantly differ between diseased and healthy animals. Lower levels of RBCs, Hb, PCV, MCV, MCH, and MCHC in PT does indicate anemia, which could be brought on by a metabolic disease that affects the production of red blood cells [69]. Furthermore, decreased erythropoiesis in pregnancy toxemia may lead to a drop in RBC and Hb levels [70]. Conversely, a significant increase in the total leucocyte, neutrophil, and lymphocyte count in PT does suggest a potential inflammatory response, possibly associated with the metabolic disorder, and may be attributed to the presence of acute and chronic inflammations [71]. Our findings aligned with previous studies [27,51,68,72], in which it was postulated that the increase was caused by infection, inflammatory responses, metabolic acidosis, and liver tissue necrosis.

In this study, does affected by pregnancy toxemia exhibited a marked reduction in glucose levels, accompanied by significant increases in serum BHB, NEFAs, urea, creatinine, AST, and ALT compared with healthy animals. Similar results have been documented by [41,42,43,44,45,50], whereas other studies reported differing outcomes [46,72], demonstrating that serum glucose concentrations were significantly higher in diseased pregnant animals than in healthy ones. According to [15], goats with PT also had hyperglycemia, elevated BHBA concentration, hypoalbuminemia, hyperglobulinemia, and hypocalcemia. Several researchers [73,74,75] anticipate hypoglycemia in the final two months of pregnancy because the fetus’s rapid growth at this point uses up the majority of the doe’s energy, which causes hypoglycemia. If the pregnant does did not receive enough carbohydrates, they would eventually develop hepatic gluconeogenesis to find an alternative to glucose for energy and lipolysis of body fat due to persistent hypoglycemia. Thus, a state of ketoacidosis will start when the affected does’ blood contains an accumulation of ketone bodies, primarily BHB and NEFAs. Overproduction of circulating NEFAs and ketone bodies, which are first conjugated in the liver and subsequently eliminated by the kidneys, occurs during the course of the disease. They damage the kidney and liver over time, infiltrate the renal tubules and hepatic cells, and cause irreversible renal damage and fatty liver formation. This explains why our research found a significant increase in the serum enzymatic activities of kidney function tests and liver enzymes. Another consequence of hypoglycemia, increased lipolysis, and hepatoneogenesis linked to pregnancy toxemia is the hyperlipidemia and hypertriglyceridemia that were obtained in the PT group.

In the present study, does affected by pregnancy toxemia exhibited a marked decrease in metabolic parameters, including total protein, albumin, and globulin. These results are consistent with earlier reports [15,45,68], but contrast with findings from the authors of [46,72], who observed no significant differences in serum total protein levels between affected and healthy animals. Furthermore, [41] elucidated a significant hypoproteinemia, hypoalbuminemia, and hyperglobulinemia in PT does. Numerous researchers have linked these changes in PT does’ proteinograms to the catabolic nature of the disease, increased protein loss from terminal renal failure associated with PT, decomposed fetuses, and increased protein degradation [47,76]. According to [75,77], the aforementioned biochemical changes were clinically translated into weakness, weight loss, immobility, fruity breath, and anxiety symptoms that would manifest later as a result of hypoglycemic encephalopathy and the production of isopropyl alcohol from acetoacetic acid.

The current study found that PT does had higher MDA and lower levels of GPx, GSH, SOD, and CAT, which together point to increased oxidative stress. Our results were in agreement with those of earlier research [27,43,78]. Reduced GPx, GSH, SOD, and CAT activity suggests that PT does’ antioxidant defense systems are weak, leaving them more vulnerable to oxidative damage. Higher lipid peroxidation in PT is indicated by the elevated MDA levels in the results, which may be a factor in cellular damage [79]. These results support the view of other researchers that oxidative stress is primarily associated with late pregnancy in sheep and is directly proportional to the number of fetuses and stage of pregnancy [80]. If this stress is not adequately managed, it may increase the risk of PT or at least make it more severe [81,82].

The inflammatory immune response is intimately linked to cytokines, which are cellular polypeptide molecules. The inflammatory response is triggered, coordinated, and terminated by them [83]. When PT does were compared to the control group, their serum levels of pro-inflammatory cytokines (IL-1α, IL-1β, IL-6, and TNF-α) significantly increased, while their levels of anti-inflammatory cytokines (IL-10) significantly decreased. Our results were consistent with earlier research [41,78,84]. According to [83,85,86], these findings are in line with earlier research that examined the physiological significance of pro-inflammatory and anti-inflammatory cytokines in pregnancy establishment, adaptation, and parturition as well as their pathological role in various pregnancy disorders like sheep pregnancy toxemia. Ref. [83] asserted that the direct cause of enhanced cytokine release is the elevated levels of circulating BHB and NEFAs associated with late gestation periods and pregnancy toxemia.

In summary, the novelty of our findings lies in demonstrating that SNPs and transcriptomic alterations, when combined with ultrasonographic evidence of fatty liver, provide a comprehensive profile of PT pathogenesis in Shami goats. To our knowledge, this is the first report integrating these approaches in this breed, offering new tools for both early clinical diagnosis and genetic selection strategies.

### Diagnostic Limitations

Firstly, an important limitation of this study is the relatively small number of pregnancy toxemia cases (*n* = 17). This reflects the practical challenges of recruiting naturally occurring clinical cases within a single breeding season. Although the limited sample size may reduce the generalizability of the findings, the consistency of hematological, biochemical, immunological, and genetic differences between affected and healthy goats strengthens the validity of the results. Future studies involving larger herds and multi-season sampling are warranted to confirm and expand these observations. Secondly, although multiple *t*-tests were performed without correction for multiple comparisons, the study was designed as an exploratory investigation to highlight potential candidate markers rather than to establish definitive cut-off values. Furthermore, while discriminant analysis achieved 100% classification accuracy, this result should be interpreted with caution given the relatively small sample size, and validation in larger, independent populations is required. Thirdly, although discriminant analysis yielded perfect classification within our dataset, this finding may be influenced by overfitting due to the limited sample size. Therefore, the apparent accuracy should not be considered definitive. Validation in larger, independent cohorts is required before these SNP markers can be applied as reliable predictive tools. Fourthly, a limited number of genes related to immunity and antioxidants were examined. Fifthly, although the increased echogenicity and parenchymal brightness observed in PT does strongly suggest hepatic fatty infiltration, histopathological confirmation was not available in this study. Future investigations should incorporate liver biopsy or postmortem examination to validate ultrasonographic findings. Finally, a limited number of genes related to immunity and antioxidants were examined. Thus, a wide range of factors has to be taken into account in subsequent research.

## 5. Conclusions

The results of this study were sufficient to achieve the stated objective by identifying molecular, genetic, and biochemical markers that distinguish pregnancy toxemia (PT) from healthy does. Pregnancy toxemia in goats is characterized by marked changes in hematological and biochemical parameters, oxidative stress, and inflammatory markers, triggering both innate and humoral immune responses. The differences in gene expression patterns (IL-6, IL-8, SOD3, HMOX1, ACACA, FASN) and the SNPs identified in this study may serve as practical biomarkers. These identified SNPs also hold potential for use in marker-assisted selection (MAS) to breed goats with resistance to pregnancy toxemia. Their application could enable early prediction and prevention of the disease, ultimately reducing long-term economic losses for animal breeders.

These markers could support early on-farm diagnosis through blood-based assays and provide a genetic basis for future studies aimed at selective breeding to improve resistance. Validation in larger populations and across breeds will be necessary before these markers can be applied in practical breeding programs. Future studies should focus on validating these findings in larger goat populations and across different breeds, as well as incorporating longitudinal monitoring to identify genetic and biochemical predictors of true resistance to PT. Functional studies will also be valuable to confirm the biological impact of the identified SNPs and their relevance for selective breeding programs.

## Figures and Tables

**Table 1:** Ingredient composition of the concentrate feed mixture (CFM).

Ingredient	Quantity
Corn	530 kg
Wheat bran	240 kg
Soya bean	230 kg
Sodium chloride	5 kg
Calcium carbonate	10 kg
Premix	1 kg
Netro-Nill	0.5 kg
Fylax	0.5 kg

**Table 2:** Primer sequences for real-time PCR of target genes (IL6, IL8, SOD3, HMOX1, ACACA, FASN, and β-actin, listed with product sizes and accession numbers).

Investigated Marker	Primer	Product Size (bp)	Annealing Temperature (°C)	GenBank Isolate	Origin
IL-6	F5′-ATGAACTCCCTCTTCACAAGCG-3′ R5′-CTACTTCATCCGAATAGCTCTCA-3′	627	60	NM_001285640.1	Current study
IL-8	F5′-CTGCTCTCTGCAGCTCTGTGTG-3′ R5′-TGGATCTTGCTTCTCAGCTCT-3′	264	58	XM_005681749.3	
SOD3	F5′-GCGGCGCTCCATGCGGTCTGCC-3′ R5′-CAGGTCGTCCTCGCCCGCGTGGA-3′	393	58	NM_001285675.1	
HMOX1	F5′-CTGGAGGAGGAGATCGAACGCA-3′ R5′-ACAGCTGGATGTTGAGCAGGAA-3′	460	58	NM_001285567.1	
ACACA	F5′-GCTGAGCTTCACACAGGCAGTC-3′ R5′-CACCACAGCCTTCATGTGTCCT-3′	477	60	DQ370054.1	
FASN	F5′-TACGCCGTGCTGGGCAGCCAGG-3′ R5′-CTCCTGAGAGATGCAGCCGTCG-3′	381	58	NM_001285629.1	
ß. actin	F5′-TGGCACCACACCTTCTACAACG-3′ R5′-GGCTTCCTTGATGTCACGGACGA-3′	390	60	AF481159.1	

IL-6 = interleukin 6, IL-8 = interleukin 8, SOD3 = superoxide dismutase 3, HMOX1 = heme oxygenase 1, ACACA = acetyl-CoA carboxylase alpha, and FASN = fatty acid synthetase.

**Table 3:** Mean values (M ± SE) of temperature, pulse, and respiration in healthy (*n* = 33) and pregnant toxemic (*n* = 17) does.

Variable	Control Group	Pregnancy Toxemic Group	*p* Value	Reference Values
Temperature (°C)	39.1 ± 0.05	37.2 ± 0.1 *	0.001	38.5–40 [37]
Pulse (beats/min)	84.3 ± 2.3	52.3 ± 1.4 *	0.001	70–90 [37]
Respiration (breaths/min)	27 ± 0.5	37 ± 0.5 *	0.001	15–30 [37]

* Values with an asterisk within the same row are statistically significant (*p* < 0.05).

**Table 4:** Immunological, antioxidant, and lipogenic marker distribution in healthy and pregnancy toxemia goats with a single base difference and possible genetic change.

Gene	SNPs	Healthy *n* = 33	Pregnancy Toxemia *n* = 17	Total *n* = 50	Chi-Square Value X2	*p* Value	Kind of Inherited Change	Amino Acid Order and Sort
IL-6	G111A	18/33	-/17	18/50	14.4	0.001	Synonymous	37 K
IL-8	T115C	21/33	-/17	21/50	18.6	0.001	Synonymous	39 L
	G196A	-/33	11/17	11/50	27.3	0.001	Non-synonymous	66 D to N
SOD3	T245C	13/33	-/17	13/50	9	0.001	Non-synonymous	82 L to P
HMOX1	T81C	17/33	-/17	17/50	13.2	0.001	Synonymous	27 A
	G309C	-/33	13/17	13/50	34.1	0.001	Synonymous	103 G
ACACA	G166C	-/33	10/17	10/50	24.2	0.001	Non-synonymous	56 G to R
	G186A	24/33	-/17	24/50	23.7	0.001	Synonymous	62 R
FASN	C48G	-/33	9/17	9/50	21.3	0.001	Synonymous	16 V
	T57C	14/33	-/17	14/50	10	0.001	Synonymous	19 S
	G214A	18/33	-/17	18/50	14.4	0.001	Non-synonymous	72 A to T

IL-6 = interleukin 6, IL-8 = interleukin 8, SOD3 = superoxide dismutase 3, HMOX1 = heme oxygenase 1, ACACA = acetyl-CoA carboxylase alpha, and FASN = fatty acid synthetase. A = alanine, D = aspartic acid, G = glycine, K = lysine, L = leucine, N = asparagine, P = proline, R = arginine, S = serine, and V = valine.

**Table 5:** Discriminant analysis for classification of type of genes and health status of examined ewes.

		Predicted Group Membership		Total
		Healthy	Diseased	
Count	Healthy	33	0	33
	Diseased	0	17	17
%	Healthy	100	0.0	100.0
	Diseased	0.0	100	100.0

**Table 6:** Mean values (M ± SE) of hematological parameters in healthy (*n* = 33) and pregnant toxemic (*n* = 17) Shami goats.

Parameter	Healthy Does	Pregnancy Toxemic Does	*p* Value
RBC (×1012/L)	10.8 ± 0.5	8.5 ± 0.4 *	0.03
Hb (g/dL)	9.8 ± 0.2	8 ± 0.3 *	0.01
PCV%	36.3 ± 0.8	30.2 ± 0.5 *	0. 007
MCV (fL)	40.1 ± 0.6	33 ± 0.5 *	0.001
MCH (pg)	9.1 ± 0.4	6.2 ± 0.1 *	0.01
MCHC (g/dL)	29.5 ± 0.4	20.8 ± 0.7 *	0.002
WBC (×109/L)	7.9 ± 0.5	10 ± 0.08 *	0.001
Neutrophil (×109/L)	6 ± 0.05	7.3 ± 0.08 *	0.003
lymphocyte (×109/L)	3.3 ± 0.1	3.9 ± 0.05 *	0.001

RBC, erythrocyte count; Hb, hemoglobin; PCV, packed cell volume; MCV, mean corpuscular volume; MCH, mean corpuscular hemoglobin; MCHC, mean corpuscular hemoglobin concentration; WBC, total leukocyte count. * Statistically significant when *p* < 0.05.

**Table 7:** Mean values (M ± SE) of biochemical parameters of healthy (*n* = 33) and pregnant toxemic (*n* = 17) does.

Parameter	Normal Does	Pregnancy Toxemic Does	*p* Value
Glucose (mg/dL)	90 ± 7.6	53.3 ± 6 *	0.02
Cholesterol (mg/dL)	70.3 ± 8.9	47 ± 7.2	0.01
Triglyceride (mg/dL)	40 ± 0.5	62 ± 1.7 *	0.003
HDL-C (mg/dL)	47 ± 7.2	31 ± 1 *	0.001
LDL-C (mg/dL)	38.2 ± 0.3	30 ± 0.5 *	0.01
Total protein P (g/dL)	7.4 ± 0.2	5.2 ± 0.3 *	0.01
Albumin (g/dL)	3.8 ± 0.1	3 ± 0.05 *	0.009
Globulin (g/dL)	3.8 ± 0.05	1.8 ± 0.05 *	0.001
Urea (mg/dL)	45 ± 0.5	64 ± 2 *	0.001
Creatinine (mg/dL)	0.6 ± 0.01	1 ± 0.03 *	0.001
NEFAs (mmol/L)	0.2 ± 0.01	0.5 ± 0.008 *	0.001
BHBA (mmol/L)	0.5 ± 0.01	2.5 ± 0.04 *	0.001
AST (U/L)	51 ± 0.5	81 ± 1.8 *	0.002
ALT (U/L)	25 ± 0.5	44 ± 1.1 *	0.001

HDL-C: high-density-lipoprotein cholesterol; LDL-C: low-density-lipoprotein cholesterol; AST: aspartate transaminase; ALT: alanine transaminase; NEFAs: non-esterified fatty acids; BHBA: betahydroxy-butyric acid. * Statistically significant when *p* < 0.05.

**Table 8:** Mean values (M ± SE) of immunological and antioxidant parameters of healthy (*n* = 33) and pregnant toxemic (*n*= 17) does.

Parameters	Healthy Does	Pregnancy Toxemic Does	*p* Value
IL1α (pg/mL)	39.7 ± 1.1	102.1 ± 1.6 *	0.001
IL 1β (pg/mL)	49.2 ± 0.5	115.4 ± 1.4 *	0.001
IL 6 (pg/mL)	28.9 ± 0.6	99.2 ± 0.3 *	0.001
IL10 (pg/mL)	133 ± 0.5	67.9 ± 0.6 *	0.001
TNFα (pg/mL)	53 ± 0.5	142.1 ± 1.2 *	0.001
GPx (U/gHb)	35.5 ± 0.3	22.2 ± 0.6 *	0.001
GSH (mg/dL)	43.3 ± 2.1	28.8 ± 0.4 *	0.001
CAT (U/mL)	35.4 ± 0.4	20.5 ± 0.4 *	0.001
SOD (U/mL)	49.3 ± 0.7	33.2 ± 1 *	0.001
MDA (nmol/mL)	5.6 ± 0.4	12.7 ± 0.6 *	0.001

IL1-α, interleukin 1 alpha; IL1-β, interleukin 1 beta; IL6, interleukin 6; TNF-α, tumor necrosis factor-alpha; IL10, interleukin 10. GPx, glutathione peroxidase; GSH, glutathione reductase; CAT, catalase; SOD, superoxide dismutase; MDA, malondialdehyde. Statistically significant difference between control group and PT is indicated by (*), when *p* < 0.05.

## Data Availability

The data will be available from the corresponding author upon request.
