# Correction: El-Sayed et al. Genomic Variants, Transcriptomic Profile, Ultrasonographic Findings, and Antioxidant and Immunological Biomarkers Linked to Pregnancy Toxemia Susceptibility in Goats. *Vet. Sci.* 2025, *12*, 891

**DOI:** 10.3390/vetsci13090986

**Published:** 2026-09-18

**Authors:** Ahmed El-Sayed, Mohamed Marzok, Huda A. Alqahtani, Amin Tahoun, Adel I. Almubarak, Rasha Yassin Elkhidr, Zakriya Al Mohamed, Elshymaa A. Abdelnaby, Hussein Babiker, Hanan M. Alharbi, Khairiah M. Alwutayd, Ahmed Ateya

**Affiliations:** 1 Department of Animal Health and Poultry, Animal and Poultry Production Division, Desert Research Center (DRC), Cairo 11753, Egypt; 2 Department of Clinical Studies, College of Veterinary Medicine, King Faisal University, Al Ahssaa 31982, Saudi Arabia; aimubarak@kfu.edu.sa (A.I.A.); relmola@kfu.edu.sa (R.Y.E.); zalmohammad@kfu.edu.sa (Z.A.M.); a.ali@kfu.edu.sa (E.A.A.); hbabiker@kfu.edu.sa (H.B.); 3 Department of Zoology, College of Science, King Saud University, P.O. Box 2455, Riyadh 11451, Saudi Arabia; hudalqahtani@ksu.edu.sa; 4 Department of Animal Medicine, Faculty of Veterinary Medicine, Kafrelshkh University, Kafrelsheikh 33516, Egypt; amtahoun@just.edu.jo or amin.abdelhady@vet.kfs.edu.eg; 5 Department of Clinical Veterinary Medical Sciences, Jordan University of Science and Technology, Irbid 22110, Jordan; 6 Department of Biology, College of Science, Princess Nourah bint Abdulrahman University, P.O. Box 84428, Riyadh 11671, Saudi Arabia; hmalharbi@pnu.edu.sa (H.M.A.); kmalwateed@pnu.edu.sa (K.M.A.); 7 Department of Development of Animal Wealth, Faculty of Veterinary Medicine, Mansoura University, Mansoura 35516, Egypt; dr_ahmedismail@mans.edu.eg

## Error in Tables

In the original publication [1], there were typographical errors in **Table 2 and Table 5**.

In **Table 2**, the PCR product size of the **β-actin** reference gene was incorrectly reported as **30 bp**. The correct PCR product size is **390 bp**. The primer sequences reported in the table were correct, and only the product length contained a typographical error.

In **Table 5**, a numerical value was inadvertently miswritten during manuscript preparation. This typographical error has now been corrected in the updated table. The corrected value is consistent with the results and interpretation presented throughout the manuscript.

These corrections are purely typographical and do not affect the analyses, results, discussion, or scientific conclusions of the study. The authors apologize for these errors. This correction was approved by the Academic Editor, and the original publication has also been updated.

## References

[1] El-Sayed A., Marzok M., Alqahtani H.A., Tahoun A., Almubarak A.I., Elkhidr R.Y., Al Mohamed Z., Abdelnaby E.A., Babiker H., Alharbi H.M. (2025). Genomic Variants, Transcriptomic Profile, Ultrasonographic Findings, and Antioxidant and Immunological Biomarkers Linked to Pregnancy Toxemia Susceptibility in Goats. Vet. Sci..

